# Preliminary Characterization of a Polycaprolactone-SurgihoneyRO Electrospun Mesh for Skin Tissue Engineering

**DOI:** 10.3390/ma15010089

**Published:** 2021-12-23

**Authors:** Enes Aslan, Cian Vyas, Joel Yupanqui Mieles, Gavin Humphreys, Carl Diver, Paulo Bartolo

**Affiliations:** 1 Department of Machine and Metal Technologies, Gumusova Vocational School, Duzce University, Duzce 81850, Turkey; enesaslan@duzce.edu.tr; 2 Department of Mechanical, Aerospace and Civil Engineering, University of Manchester, Oxford Road, Manchester M13 9PL, UK; cian.vyas@manchester.ac.uk (C.V.); joel.yupanquimieles@manchester.ac.uk (J.Y.M.); 3 School of Health Sciences, University of Manchester, Oxford Road, Manchester M13 9PL, UK; gavin.humphreys@manchester.ac.uk; 4 Department of Engineering, Manchester Metropolitan University, Manchester M15 6BH, UK; c.diver@mmu.ac.uk; 5 Singapore Centre for 3D Printing, School of Mechanical and Aerospace Engineering, Nanyang Technological University, Singapore 639798, Singapore

**Keywords:** electrospinning, honey, polycaprolactone, skin

## Abstract

Skin is a hierarchical and multi-cellular organ exposed to the external environment with a key protective and regulatory role. Wounds caused by disease and trauma can lead to a loss of function, which can be debilitating and even cause death. Accelerating the natural skin healing process and minimizing the risk of infection is a clinical challenge. Electrospinning is a key technology in the development of wound dressings and skin substitutes as it enables extracellular matrix-mimicking fibrous structures and delivery of bioactive materials. Honey is a promising biomaterial for use in skin tissue engineering applications and has antimicrobial properties and potential tissue regenerative properties. This preliminary study investigates a solution electrospun composite nanofibrous mesh based on polycaprolactone and a medical grade honey, SurgihoneyRO. The processing conditions were optimized and assessed by scanning electron microscopy to fabricate meshes with uniform fiber diameters and minimal presence of beads. The chemistry of the composite meshes was examined using Fourier transform infrared spectroscopy and X-ray photon spectroscopy showing incorporation of honey into the polymer matrix. Meshes incorporating honey had lower mechanical properties due to lower polymer content but were more hydrophilic, resulting in an increase in swelling and an accelerated degradation profile. The biocompatibility of the meshes was assessed using human dermal fibroblasts and adipose-derived stem cells, which showed comparable or higher cell metabolic activity and viability for SurgihoneyRO-containing meshes compared to polycaprolactone only meshes. The meshes showed no antibacterial properties in a disk diffusion test due to a lack of hydrogen peroxide production and release. The developed polycaprolactone-honey nanofibrous meshes have potential for use in skin applications.

## 1. Introduction

The role of skin, a complex and multi-layered organ covering the outside of the body, is crucial in bodily protection, regulation, and sensorial capabilities. Trauma, disease, chemicals and radiation, and burns can lead to serious damage to skin function and appearance. The natural wound healing capacity of skin is a complex biological process to repair and regenerate the skin structure when an injury occurs [1,2,3,4]. Normally this occurs through a sequence of integrated and overlapping events. This is comprised of hemostasis (platelet activations), inflammation (cytokines and growth factors released by neutrophils and monocytes/macrophages), migration and proliferation (increasing the number of fibroblasts, endothelial cells, and keratinocytes in the wound bed to induce and manage extracellular matrix biosynthesis, epithelialization, and angiogenesis), and maturation (remodeling the tissue and collagen type changes III to I) [1,4,5]. The extracellular matrix (ECM), the biological scaffold of tissues and organs that defines the cellular 3D environment, interacts with cells to mediate proliferation, migration, and differentiation [6,7,8,9,10]. This interactive relationship between cells, biomolecules (e.g., growth factors), and the ECM components induces the tissue to recover integrity after a wound [3,4]. However, when this sequence is negatively impacted due to disease (e.g., diabetes), burns, and trauma, this can result in the healing process not occurring in a timely manner. Subsequently, chronic wounds can develop and the natural healing process no longer functions correctly [11,12].

Wound management, chronic wounds, and burns are key challenges for healthcare systems across the world both from a clinical and financial perspective. Approximately 3.8 million people with a wound were treated by the National Health Service (NHS, United Kingdom) in 2017/2018 with an annual cost of £8.3 billion [13]. For example, the average cost of treating a burn over a 24-month period is approximately £17,000 in the NHS [14].

Besides these costs, which pose a serious economic burden to healthcare systems worldwide, societal costs due to loss of economic activity must be also taken into consideration. Furthermore, the pain and discomfort cause significant distress to patients and unsuccessful treatment can lead to further serious complications such as amputation and even death. Additionally, bacterial colonization of wounds and increasing antibiotic resistance, estimated to cause up to 10 million deaths per year by 2050, causes further difficulties in managing wounds [15,16,17,18]. Therefore, cost-effective and efficacious wound treatment strategies are critical.

Appropriate clinical management of chronic wounds and burns is crucial for successful healing. An important process is debridement, the cleaning and removal of non-viable tissue, which can be followed by the use of wound dressings to protect and promote healing at the wound site [11,12]. Wound dressings such as gauze, films, hydrocolloids, foams, and alginates are commercially available and used clinically as they can absorb exudate and provide a moist environment. Antimicrobial agents can be incorporated into these dressings such as antibiotics and silver nanoparticles to prevent bacterial colonization, although the presence of biofilms in the wound can limit their efficacy [19]. Large-scale skin defects arising from surgery or burns are currently treated using the clinical gold standard of skin auto- and allografts [20,21]. However, these approaches have limitations such as the potential for donor site morbidity, limited availability of tissue, long hospitalization times, and the risk of disease transmission and tissue rejection in allografts [21]. To overcome these limitations, advanced skin substitutes and wound dressings have been developed utilizing the principles of tissue engineering and technologies such as electrospinning and bioprinting [22,23,24].

Electrospinning is a flexible manufacturing technology to fabricate nano- and microfibers, these are advantageous in the development of skin and wound dressings applications [2,25,26]. The nanofibrous meshes mimic the scale of the native ECM, have high surface to volume ratios, and high porosity which allows cell attachment and proliferation, vascularization, and oxygen and nutrient exchange [27]. Furthermore, the wide variety of materials (natural and synthetic) and solvents that can be utilized in electrospinning allows a range of applications to be considered. Subsequently, various material compositions have been explored for delivery drugs [28,29,30], antimicrobial agents [31,32,33,34,35,36,37,38], chemotherapy [39,40,41], and to promote tissue regeneration [42,43,44,45,46,47]. For example, Movahedi et al. [46] have produced electrospun aspirin-loaded polycaprolactone (PCL) and maltodextrin composite meshes and observed that the inclusion of aspirin presented better cell adhesion and proliferation in vitro, and faster wound closure in vivo. Alternatively, metallic particles such as silver can be utilized for their antibacterial properties, but also to promote tissue regeneration, for example, Adhikari et al. [47] have electrospun PCL with magnesium particles, which promoted enhanced cell proliferation, vascularization, and healing. Natural materials have also been investigated for their antibacterial attributes and as wound dressings such as using *Chelidonium majus* L. extract combined with PCL, polyvinyl alcohol, and pectin electrospun meshes [38]. The meshes inhibited growth of *Staphylococcus aureus* and *Pseudomonas aeruginosa* with no cytotoxic effects on human dermal fibroblasts. The versatility of electrospinning is attractive for wound dressings and pharmaceuticals as the mesh can provide localized delivery with a controlled release profile, thus decreasing systemic side-effects and improving efficacy [48].

Antimicrobial agents have been incorporated into electrospun dressings; however, there are issues arising from growing antibiotic resistance and potential cytotoxicity associated with the use of heavy metals such as silver nanoparticles [17,49]. Therefore, broad-spectrum antimicrobials are required that avoid these specific issues. Honey is an example of a material that has been used to treat wounds since ancient times due to its broad-spectrum antimicrobial properties [50,51]. This is due to the production of hydrogen peroxide (H_2_O_2)_, high osmolality, and low pH [51,52,53]. Additionally, reactive oxygen species such as H_2_O_2_ can stimulate wound healing by modulating and guiding cellular repair processes, for example in tail regeneration in tadpoles [54,55]. Subsequently, honey has been explored for use in wound dressings and tissue engineering applications [56,57]. For example, honey has been incorporated into an electrospun nanofibrous mesh of polyvinyl alcohol and alginate [35]. Higher concentrations of honey loading showed antibacterial properties against both gram-negative and positive bacteria. Furthermore, manuka honey and silk fibroin electrospun meshes have been developed, which showed high cell viability, antibacterial properties, and wound healing in a mouse model comparable to a commercial dressing [36]. Additionally, manuka honey has been electrospun with polycaprolactone (PCL) and shown to have significant antibacterial properties against gram-negative bacteria [37]. The presence of methylglyoxal in manuka honey is the main non-peroxide mode of antibacterial action. Commercial non-medical grade honey sources can have variations in composition and sterility making them not suitable for biomedical applications. Medical grade and engineered honey such as SurgihoneyRO™ (SH) are a suitable source to incorporate into wound dressings and skin tissue engineering constructs. SH has been shown to have controlled and prolonged production of H_2_O_2_ with broad-spectrum antimicrobial action and antibiofilm properties without the use of additional antibiotics or non-peroxide mechanisms of action such as methylglyoxal [52,53,58]. Furthermore, application of SH aided healing in a range of wounds including trauma, leg ulcers, surgical wounds, and diabetic foot ulcers [59].

This preliminary study investigates the development of a composite electrospun mesh composed of PCL, a biocompatible and degradable synthetic polymer widely used in electrospinning, and medical grade SH for use in wound dressings and skin tissue engineering applications. Previously, we have shown that electrospun PCL and SH meshes are cytocompatible [60]. However, this study further optimizes the electrospinning process and comprehensively characterizes mesh morphology, topography, chemistry, and mechanics. This detailed material characterization is required in the translation and development of biomedical devices. Furthermore, initial studies of cell biocompatibility and antibacterial properties as a function of SH concentration are performed. This preliminary study demonstrates the successful fabrication of an electrospun mesh with uniform nanofibers at different SH concentrations, which have a positive impact on cell attachment and proliferation.

## 2. Materials and Methods

### 2.1. Material Preparation

PCL (Mw 50,000 g/mol; Perstorp Caprolactones, Cheshire, UK) and SH (Matoke Holdings, Abingdon, UK) solutions were prepared using acetic acid (Fisher Scientific, UK) as the solvent. Four different PCLSH compositions were prepared: 0, 10, 20, and 30% *w/w* of SH, termed PCLSH10, PCLSH20, PCLSH30, respectively. Briefly, PCL was dissolved in 10 mL of acetic acid and stirred overnight using a hot plate magnetic stirrer at 40 °C. The solution was allowed to cool to room temperature before addition of SH and further stirring (~15 min) until a homogenous solution was obtained. For example, PCLSH10 consists of 18 w/v% PCL and 2 w/v% SH.

### 2.2. Electrospun Mesh Fabrication

A vertical collecting electrospinning system (Prefector, Spraybase, Maynooth, Ireland) was used to produce nanofibrous meshes and consists of a high voltage power supply (0–30 kV), syringe pump, control software, stainless steel collector, and a 18G × 25 mm stainless steel emitter (needle). A comprehensive optimization study was conducted to select optimal processing parameters to fabricate the meshes. Three different voltages (10, 14, and 17 kV), five different flow rates (0.0083, 0.0125, 0.0166, 0.05, and 0.1 mL/min.), and three different collector-needle distances (10, 13.7, and 18.7 cm) were considered. Electrospun nanofibers were collected on aluminum foil for 5 min for the initial optimization study. Optimal processing parameters were selected based on scanning electron microscopy images using fiber uniformity, distribution, and absence of beads as selection criteria (Figures S1–S4). The parameters selected for fabrication of electrospun meshes and experimental analysis was a voltage of 17 kV, 0.1 mL/min flow rate, 18.7 cm collector-needle distance, and 30 min collection time. The electrospun meshes were cut into circles (diameter: 16 mm) for all studies; apart from mechanical testing, degradation and swelling, and antibacterial, and finally placed in a vacuum chamber overnight.

### 2.3. Morphological and Topographical Analysis

Electrospun mesh morphology was characterized using a scanning electron microscopy (SEM; Hitachi S-3000N, Tokyo, Japan). Meshes were sputter coated with gold (~10 nm thickness) and imaged with an accelerating voltage of 15 kV. Fiji software with the DiameterJ plugin was used to analyze fiber diameter and distribution [61].

The surface topography of electrospun meshes was investigated using white light interferometry (WLI; Contour GT-K, Veeco Bruker, Billerica, MA, USA) equipped with Vision64 analytical software (Veeco Bruker, Billerica, USA). The samples were scanned with 10 µm as a backscan and 40 µm as a length with a 5% threshold and processed considering vertical scanning interferometry method. The surface images were taken using 115x objective lens and a 2x zoom from the surface. The root mean square roughness (S_q_) was calculated for each electrospun mesh (*n* = 5) with at least three different positions on the mesh. Sq was considered as the parameter provides more precise values than S_a_ [62].

### 2.4. X-ray Diffraction

The structure and crystallinity of the electrospun meshes was investigated using X-ray diffraction (XRD, ProtoXRD, Proto Manufacturing, Taylor, MI, USA). A CuKα radiation source and wavelength of λ = 1.54060 Å in the 2θ range between 0° and 50° with a step size of 0.02° was used. The applied voltage was 40 kV and the current was 40 mA. The percentage of crystallinity was approximated by dividing the total area of crystalline peaks (2θ: 16–25.8°) by the total area (2θ: 10–49.9°) under the XRD pattern [63,64].

### 2.5. Fourier Transform Infrared and X-ray Photoelectron Spectroscopy

The material chemistry of the PCLSH electrospun meshes was investigated using Fourier-transform infrared spectroscopy (FTIR, Alpha-P System, Bruker, Coventry, UK) and X-ray photoelectron spectroscopy (XPS, Axis Ultra Hybrid, Kratos Analytical, Manchester, UK). The FTIR transmittance spectra was collected between 4000 cm^−1^ to 400 cm^−1^ using a 24-scan resolution at 4 cm^−1^. The XPS was run at 15 mA and emission at 225 W. The pass energies of low and high resolution were 80 and 20 eV, respectively.

### 2.6. Wettability

The wettability of meshes (*n* = 5) was determined through static contact angle measurement (KSV Cam 200, KSV Instruments, Espoo, Finland). Images were obtained at 0 and 50 s after droplet formation and subsequently analyzed using the Sessile drop technique.

### 2.7. Tensile Mechanical Characterisation

The mechanical properties of the electrospun meshes were investigated using tensile testing (Instron 3344, Instron, Norwood, MA, USA) and associated software (Bluehill Universal Software, Instron, Norwood, MA, USA) for analysis following the ISO-527 standards. Electrospun meshes (*n* = 6) were removed from the foil and cut into strips (5 mm × 25 mm), clamped in the center of the machine, and stretched in the dry state at a deformation rate of 5 mm/min using a 10N load cell until break. The Young’s modulus was determined using the slope of the stress-strain curves in the initial linear regime within the range of 1–3% strain.

### 2.8. Degradation and Swelling

The degradation and swelling of the meshes were assessed using two different media, Dulbecco’s Modified Eagle Medium (DMEM) and simulated body fluid (SBF), which was prepared following Kokubo and Takadama’s protocol [65]. Meshes (*n* = 3) were cut into 1 × 1 cm samples and placed into 24-well plates with either DMEM or SBF and statically incubated at 37 °C. The experiment was conducted for 63 days and the DMEM and SBF were replaced once a week. The same samples (*n* = 3) were used to measure the wet weight (W_w_) during the first three days of the swelling study (timepoints: 0, 1, 4, 18, 24, 48, and 72 h). After this, different samples (*n* = 3) were used to complete the swelling and degradation studies considering the following time points: days 3, 7, 14, 21, 35, 49, and 63. At each timepoint, samples were removed from the media and the weights of the wet electrospun meshes (W_w_) measured. The swelling (S_w_) was calculated according to the following equation:

Sw = W_w_/W_i_ (1)

where W_i_ is the initial dry weight and W_w_ is the weight of the wet electrospun mesh. Subsequently, the swollen meshes were dried in the incubator at 37 °C for 10 days and weighed again to calculate the dry weight (W_d_). The percentage of weight loss (Dl) was calculated as follows:

Dl = ((W_i_ − W_d_)/W_i_) ∗ 100 (2)

### 2.9. Cell Metabolic Activity and Viability

Electrospun meshes were biologically assessed using human dermal fibroblasts (HDFs) (Sigma Aldrich, UK) and human adipose-derived stem cells (hADSCs) (STEMPRO^™^, Thermo Fisher Scientific, Waltham, USA). HDFs were cultured (37° C, 5% CO_2_, and 95% humidity) with DMEM containing 10% (*v*/*v*) fetal bovine serum, 1% (*v*/*v*) glutamine, and 1% (*v*/*v*) penicillin/streptomycin until 80% confluence and harvested using 0.05% trypsin-EDTA solution (Thermo Fisher Scientific, Waltham, MA, USA) at passage 2. A total of 15,000 cells in 150 µL of media were seeded onto each mesh and incubated for 4 h to allow cell attachment, before the addition of 350 μL fresh media. hADSCs cells were cultured with MesenPRO RS™ media containing 2% (*v*/*v*) growth supplement, 1% (*v*/*v*) glutamine, and 1% (*v*/*v*) penicillin/streptomycin until 80% confluence and harvested using 0.05% trypsin-EDTA solution (Thermo Fisher Scientific, Waltham, USA) at passage 7. A total of 50,000 cells in 150 µL of media were seeded onto each mesh and incubated in a cell culture incubator (37 °C, 5% CO_2_, and 95% humidity) for 4 h to allow cell attachment, before the addition of 450 μL fresh media. For both cases, prior to cell seeding the meshes were sterilized using 80% ethanol for 2 h and then dried overnight in a sterile laminar flow cabinet.

The metabolic activity of cells, an indicator of cell proliferation, was evaluated using the resazurin assay (Alamar Blue) (Sigma-Aldrich, Gillingham, UK). The fluorescence intensity was measured on days 1, 3, 7, and 14 after cell seeding (*n* = 4 for HDF and *n* = 7 for hADSCs). On day 1, all samples were transferred to a new 24-well plate to prevent unattached cells from influencing the result. At each time point, 10% of the total media by volume (50 µL for HDF and 60 µL for hADSCs) of resazurin solution (0.01% (*v*/*v*)) was added to each sample and incubated for 4 h. After incubation, 150 µL of each sample was transferred to a 96-well plate and the fluorescence intensity was measured (540 nm excitation/590 nm emission wavelength) with a plate reader (infinite 200, Tecan, Männedorf, Switzerland). Samples were washed twice in sterile PBS to remove the resazurin solution before the addition of fresh media. Cell culture media was changed every 3 days.

Cell viability was assessed using a Live/Dead Assay Kit (ThermoFisher Scientific, Loughborough, UK) at day 3 and day 14 for HDFs, and at day 1 and day 14 for hADSCs according to the manufacturer’s instructions. Cell culture media was removed from the samples and were washed with PBS twice before adding 500 µL of calcein-AM (2 µM) and EthD-1 (4 µM) to the PBS solution. The samples were then incubated for 25 min prior to imaging with a confocal fluorescence microscope (Leica TCS SP8, Leica Microsystems, Wetzlar, Germany).

### 2.10. Glucose and Hydrogen Peroxide Concentration

Peroxidase from horseradish (HRP) (Type VI, lyophilized powder, ≥250 units/mg), sodium acetate buffer solution (pH:5.2, 3 M), ABTS™ (2,2′-Azino-bis(3-ethylbenzothiazoline-6-sulfonic acid), and H_2_O_2_ solution (30% *w/w* in H_2_O) were all purchased from Sigma-Aldrich, UK; D(+)-Glucose Anhydrous (180.16 g/mol) was purchased from ThermoFisher Scientific, UK.

A reaction buffer was prepared by diluting sodium acetate buffer solution in distilled water to 30 mM. A total of 50 mM glucose, 5 mM ABTS, 10 U/mL HRP, and 15.82 U/mL glucose oxidase stock solutions were then prepared using the 30 mM buffer. Working solutions were prepared by mixing 10 mL of 5 mM ABTS with 10 mL of 10 U/mL HRP. All stock solutions were used on the same day they were prepared. Glucose and H_2_O_2_ standard curves were prepared by diluting glucose and H_2_O_2_ stock solutions to concentrations of 0 to 200 μM, and 0 to 400 μM, respectively.

In this initial study, only PCL and PCLSH30 electrospun meshes (*n* = 3) were considered at timepoints of 0, 2, 6, 24, and 48 h. Meshes were placed in a 24-well plate and 500 µL of 30 mM sodium acetate buffer solution was added, plates were kept at room temperature and protected from light. A total of 100 µL were taken from each well (and replaced immediately with buffer) at each timepoint and loaded into a 48-well plate. A total of 200 µL of the working solution and 100 µL of 15.82 U/mL GOx solution were added to each microplate well and allowed to incubate for 15 min at room temperature. Absorbance was measured at 420 nm using a plate reader (CLARIOstar, BMG Labtech, Ortenberg, Germany).

### 2.11. Antibacterial Analysis

Prior to mesh production, the collector and the aluminum foil were sterilized with 80% ethanol. The electrospun meshes (*n* = 6) were cut using a sterile punch into 6 mm diameter circles for disk diffusion antibacterial testing. The meshes were sterilized using ultraviolet (UV) light for 30 min on both sides.

Prior to disk diffusion tests, mannitol salt agar (MSA; Sigma Aldrich, Gillingham, UK) plates were prepared following the manufacturer’s instructions The MSA solution was sterilized in an autoclave for 2 h at 121 °C, then kept at 55 °C to prevent solidification until pouring into Petri dishes and left at room temperature to solidify. A Mueller-Hinton broth (MHB; Sigma Aldrich, Gillingham, UK) solution was prepared following the recommended manufacturers protocol. The solution was sterilized by autoclaving at 121 °C for 2 h, stored at room temperature, and opened under a Bunsen flame.

The ASTM E3161-18 standard operating procedure was followed to culture *Staphylococcus aureus* (*S.aureus*-Gram-positive), *Pseudomonas aeruginosa* (*P. aeruginosa*-Gram-negative), and *Escherichia coli* (*E. coli*-Gram-negative). Defrosted bacterial strain was added to 10 mL of MHB and vortexed to obtain a homogenous solution and incubated overnight at standard conditions (37 °C and 5% CO_2_). The following day, the absorbance of the bacterial solution was measured using an optical spectrometer to guarantee an optical density of 0.08, which is equivalent to 0.5 McFarland standard for disk diffusion method. The bacterial solution was spread on the agar plate, followed by placing the meshes on the agar plates (four meshes per plate). The agar plates were labelled and placed in the incubator overnight at 37 °C. Finally, the inhibition zone was measured to obtain the effect of the electrospun meshes on the different bacterial strains.

### 2.12. Statistical Analysis

Statistical analysis was performed using one-way analysis of variance (ANOVA) and post-hoc Tukey test using GraphPad Prism 7 software (Graphpad Software Inc., San Diego, CA, USA). A *p* value < 0.05 (* = *p* < 0.05, ** = *p* < 0.01, *** = *p* < 0.001, **** = *p* < 0.0001) was considered statistically significant and results are reported as mean ± standard deviation (SD).

## 3. Results and Discussion

### 3.1. Morphology and Topography

**Figure 1:**
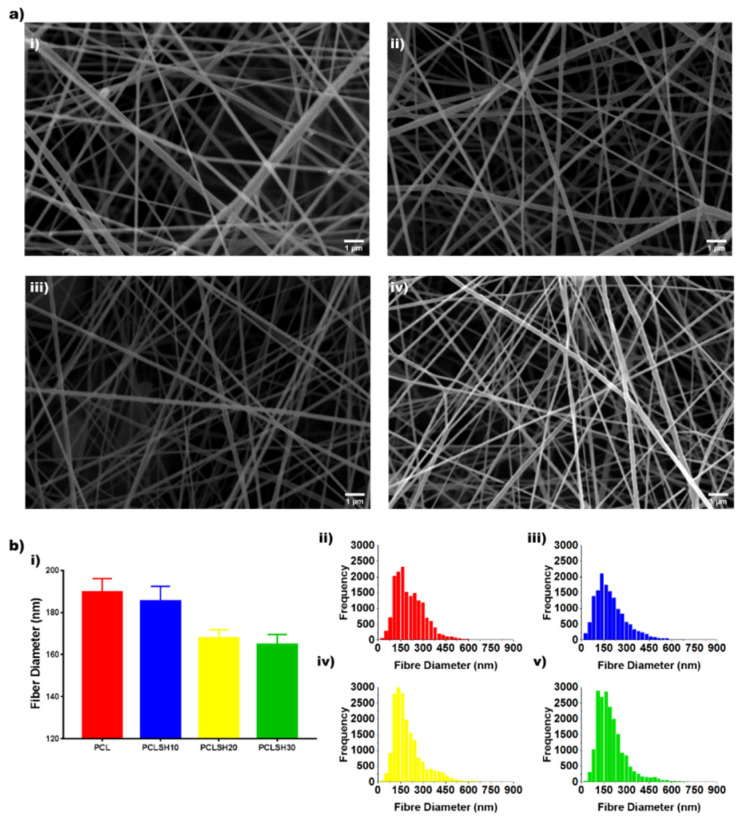
(**a**) SEM images of electrospun meshes: (**i**) PCL, (**ii**) PCLSH10, (**iii**) PCLSH20, and (**iv**) PCLSH30 (scale = 1 µm). (**b**) (**i**) Mean fiber diameter and distribution of fiber diameters for (**ii**) PCL, (**iii**) PCLSH10, (**iv**) PCLSH20, and (**v**) PCLSH30 meshes.

The electrospun PCL and PCLSH meshes morphology were imaged using SEM (Figure 1a). Nanofibrous meshes with a random orientation were successfully produced with nanoscale fibers mimicking the native tissue ECM. The optimized parameters used show no formation of beads. The results show that the mean fiber diameter decreases by increasing the SH concentration (PCL:190 nm > PCLSH30: 165 nm) due to the reduced content of PCL (Figure 1b). The electrospun meshes exhibit a uniform distribution profile of fiber diameters ranging from 50 nm to 600 nm, with higher concentrations of SH having a narrower profile.

**Figure 2:**
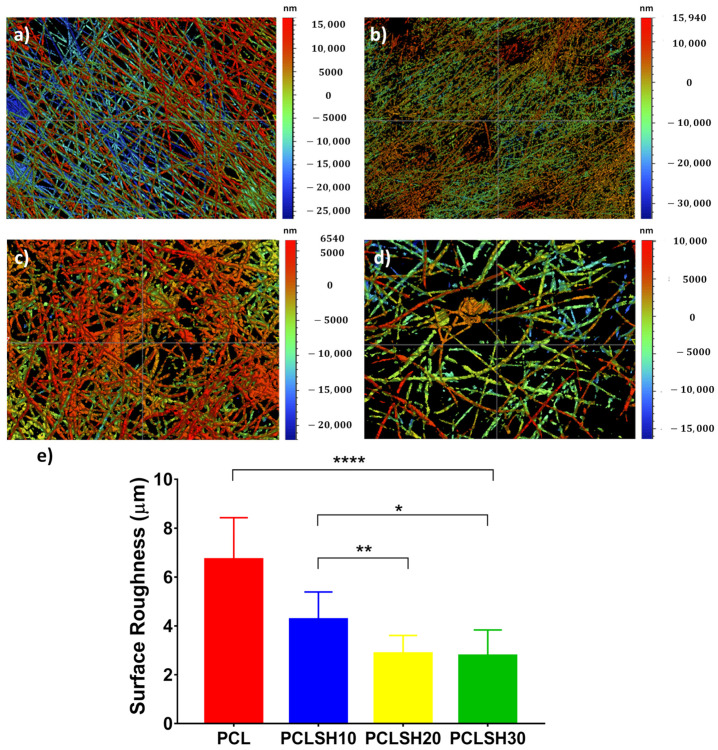
WLI images of the surface topography of the electrospun meshes (**a**) PCL, (**b**) PCLSH10, (**c**) PCLSH20, and (**d**) PCLSH30 meshes; and (**e**) surface roughness of meshes.

The surface topography of the electrospun meshes was observed using WLI (Figure 2). The results show that the surface roughness decreases with increasing SH concentration. PCL has the highest surface roughness (6.7 µm), whilst PCLSH30 has the lowest (2.77 µm). This can be related to the fiber diameter, which decreases with increasing SH, and a more uniform fiber distribution observed in meshes containing high levels of SH, as the surface roughness was calculated for the entire meshes and not individual fibers.

### 3.2. Mesh Crystallinity

**Figure 3:**
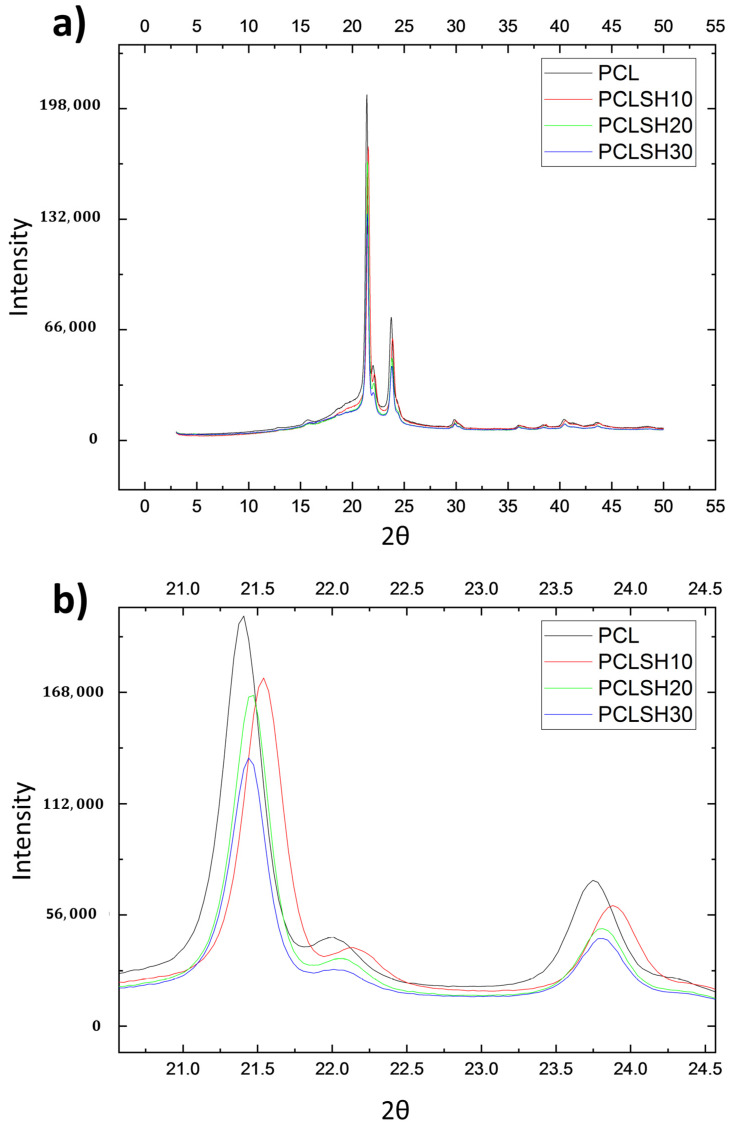
XRD patterns of electrospun meshes. (**a**) General view and (**b**) magnified image of the peaks for all samples.

The crystallinity of the electrospun meshes was observed using XRD (Figure 3). The results show that increasing the SH concentration in the electrospun meshes did not affect the characteristic diffraction peaks of PCL, 2θ = 21.40° and 23.74° corresponding to the (110) and (200) planes of the orthorhombic crystal lattice structure, respectively [66,67]. Rather the peak area and peak position changes (Figure 3b). This can be attributed to changes in crystallization and microstructure due to the addition of SH [63,64]. The SH decreases the polymer crystallinity, the peak area decreases, from 64% (PCL meshes) to 59% (PCLSH30 meshes).

### 3.3. Chemical Analysis

**Figure 4:**
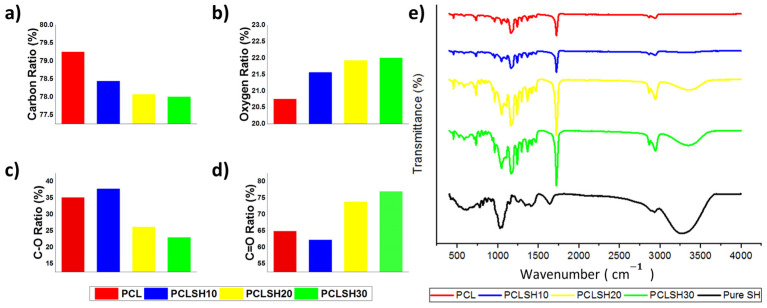
Electrospun mesh (**a**) carbon, (**b**) oxygen, (**c**) C-O, (**d**) C=O percentage ratios, and (**e**) FTIR spectrum.

XPS and FTIR analysis allow the determination of changes in chemistry in the electrospun meshes due to the incorporation of SH. XPS shows that the ratio of carbon (C) and oxygen (O) in the meshes changes due to inclusion of SH (Figure 4a–d). PCL exhibits 79.25% C and 20.75% O, whilst the PCLSH30 mesh has 78% C and 22% O. These results indicate an increase in the amount of O in SH containing meshes and as a result the C:O ratio decreases. The bond type (C-O and C=O) in the electrospun meshes showed that the PCL meshes presented 65% C=O and 35% C-O, while by increasing the level of SH in the meshes the percentage of C=O bonds increases to 77% (PCLSH30), while the percentage of C-O bonds decreases to 23%.

The FTIR spectrum shows that the key characteristic peaks of SH are present in the composite PCLSH meshes (Figure 4e). Increasing concentration of SH results in an increase in the maximum peak intensity observed in the spectrum between 920 and 1550 cm^−1^. In addition, between the range of 920 and 1550 cm^−1^ the bands for C-O stretching, C-H bending, -OH bending vibrations, C(O)-O stretching vibrations, and –C-O-H in-plane bending vibrations can be observed for both SH and PCL. The molecular interactions between the SH and PCL components potentially increase the intensity of the bands in the spectrum. A similar impact can be seen between 3000–3506 cm^−1^ as a result of the stretching vibration of –O-H groups [68]. The intense peak at 1721 cm^−1^ is due to the presence of carbonyl groups (C=O stretching), which are associated with PCL [34]. Moreover, the intensity of this peak increases with increasing SH concentration due to the hydroxyl (OH) groups present in the SH interacting with the carbonyl (C=O) groups present in PCL. This supports the increase in the C=O ratio with increasing concentration of SH in the meshes. The XPS and FTIR results confirm the incorporation of SH into the PCL meshes.

### 3.4. Mesh Wettability

**Figure 5:**
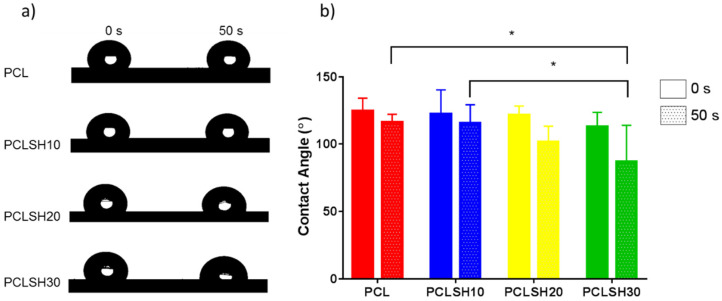
Wettability of electrospun meshes. (**a**) Droplet on the mesh surfaces and (**b**) contact angle measurements at 0 s and 50 s.

The wettability of the meshes as measured by water contact angle shows immediately after droplet formation all meshes present a hydrophobic surface (Figure 5). PCL exhibits the highest contact angle (124.6°) and PCLSH30 the lowest one (112.9°). After 50 s, the contact angle for all meshes decreases. PCLSH30 decrease is the largest and has a contact angle of 86.8°, while the contact angle of PCL meshes is 116.1°. The contact angle decreases with increasing SH concentration. This indicates that the addition of SH enhances the hydrophilicity of the electrospun meshes. This is due to the changes in the chemical structure, presence of hydroxyl groups, and morphology of the meshes.

### 3.5. Mechanical Analysis

**Figure 6:**
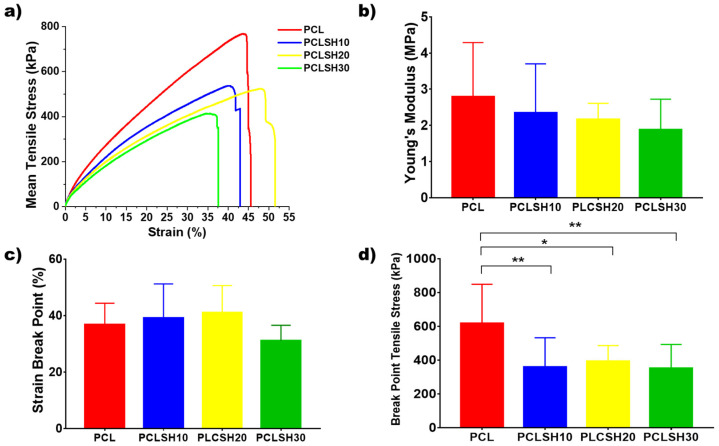
(**a**) Representative tensile stress–strain curves, (**b**) Young’s modulus, (**c**) percentage strain break point, and (**d**) tensile stress at break point for the electrospun meshes.

The tensile mechanical properties of the electrospun meshes were tested to assess their suitability for skin tissue engineering applications (Figure 6). The stress–strain curves show an initial linear elastic region before rupture of the meshes between 35–55% strain (Figure 6a). The tensile Young’s modulus and ultimate tensile strength of the meshes decreases by increasing the SH concentration. This is due to the decrease in PCL concentration in the composite and, consequently, a decrease in the fiber diameter.

The PCL meshes present the highest Young’s modulus value (4.42 ± 1.77 MPa) due to the larger fiber diameter and higher crystallinity. The Young’s modulus of the PCLSH meshes decreases with increasing SH concentration from 2.34 to 1.87 MPa for PCLSH10 and PCLSH30, respectively (Figure 6b). The lower PCL content, the reduction in crystallinity of the meshes, and decrease in fiber diameter all contribute to the decrease in mechanics due to the addition of SH. These results indicate that all meshes may be suitable for skin tissue engineering applications as they have a Young’s modulus within the range of skin tissue, which can vary between 5 kPa and 140 MPa [69].

The SH containing meshes have a strain break point of 43, 52, and 38%; and break point tensile stress of 359 ± 173 kPa, 393 ± 93 kPa, and 351 ± 141 kPa for PCLSH10, PCLSH20, and PCLSH30, respectively (Figure 6c,d). PCL has similar strain break point, but significantly higher break point tensile stress compared to SH containing meshes. However, the slightly lower values for PCLSH30 can potentially be explained due to the lower fiber diameter and the increase in the level of functional groups (C=, OH), which have been reported to have a negative impact on elasticity [70].

### 3.6. Swelling and Degradation

**Figure 7:**
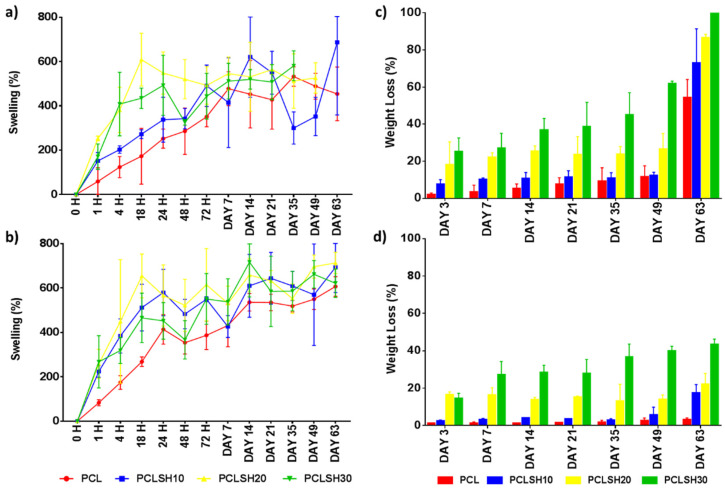
Swelling in (**a**) SBF and (**b**) DMEM and degradation in (**c**) SBF and (**d**) DMEM of the electrospun meshes as a function of time.

The swelling and degradation profiles for the PCL and PCLSH electrospun meshes are presented in Figure 7. All samples reached a swelling equilibrium state after day 7 and 14 in SBF and DMEM solution, respectively. The meshes in DMEM swelled more than in SBF. This is due to the meshes starting to degrade earlier in SBF solution than in DMEM. Furthermore, meshes containing SH swelled more than PCL only due to the higher hydrophilicity of the SH meshes. Hydroxyl groups in water form hydrogen bonds with other hydroxyl groups present in SH and consequently the liquid absorption is higher in PCLSH meshes than pure PCL meshes. Consequently, the ability to swell and absorb water is advantageous in wound dressing applications as wound exudate requires management.

The PCL meshes present no degradation in DMEM, while losing approximately half of their weight in SBF liquid at day 63 (Figure 7c,d). Conversely, the SH-containing meshes have an accelerated degradation profile in both media. The increase in SH content increases the degradation rate due to the water solubility of SH. The results show that there is a faster degradation in SBF than DMEM. The PCLSH30 meshes completely degrade by day 63 in SBF and the PCLSH20 meshes loses 86% of their weight. The degradation profile in DMEM by day 63 is similar to the percentage weight of SH in the mesh, indicating that only the SH has leached out of the mesh and PCL remains. All meshes, apart from PCLSH20 and PCLSH30 samples immersed in SBF, remained intact up to day 63 and could be handled. SEM imaging is required throughout this period to assess changes in fiber morphology and allow assessment of their use in wound dressing applications. The difference in degradation behavior between the samples immersed in SBF and DMEM with PCL degrading faster in SBF is unclear. In both solutions, the PCL should undergo a similar hydrolytic degradation pathway, with amorphous regions degraded initially followed by crystalline regions [71,72]. A study of PCL-tricalcium phosphate thin film scaffolds showed a slight increase in degradation in DMEM compared to SBF [73]. However, the processing and scaffold morphology are significantly different to the PCLSH electrospun mesh, thus comparison is difficult. Further investigation is required to understand how scaffold composition and morphology effects degradation behavior in different media and how the media composition mediates this degradation profile.

Finally, as SBF mimics the body fluids, these results are promising as they show that PCLSH meshes could have an accelerated degradation profile in vivo. However, further investigation is required as the degradation process in vitro using SBF and the weekly change of media is different to the actual environment in vivo.

### 3.7. Cell Metabolic Activity and Viability

**Figure 8:**
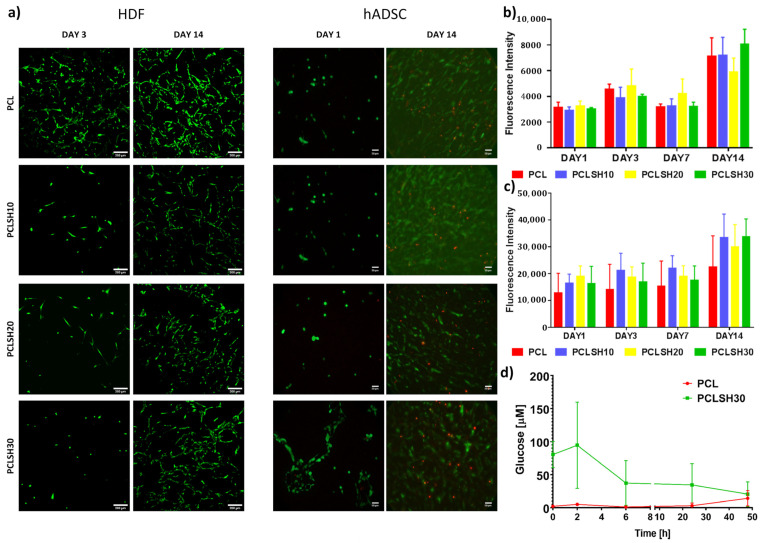
Cell metabolic activity and viability of HDFs and hADSCs on the electrospun meshes. (**a**) HDF viability at days 3 and 14 (green = live, red = dead) (scale = 200 µm) and hADSC viability at day 1 and 14 (scale = 50 µm). Metabolic activity of (**b**) HDFs and (**c**) hADSCs at day 1, 3, 7, and 14. (**d**) Glucose content of PCL and PCLSH30 meshes up to 48 h.

The PCLSH electrospun meshes’ biocompatibility was assessed through seeding of HDFs and hADSCs, and evaluating cell metabolic activity and viability for up to 14 days (Figure 8). Cell seeding efficiency for both cell types on day 1 was over 55% for all electrospun meshes.

Cell viability was high, ~95%, in all meshes at the initial timepoint, day 1 and 3, for hADSCs and HDFs, respectively, and high viability was maintained till day 14 (Figure 8a). A few dead hADSCs were observed at day 14, potentially due to the high cell density. At day 3, the HDF cells attached to the PCLSH meshes have a less spread and rounder morphology than PCL only. A round morphology for hADSCs was seen on all electrospun meshes at day 1, apart from PCLSH30, which had a slightly more elongated shape. Although the PCLSH meshes are more hydrophilic, this may not be significant enough to alter cell attachment. Furthermore, protein adsorption onto the fibers may be altered due to the presence of SH which also influences cell attachment and spreading. Additionally, the lower surface roughness of the PCLSH meshes may also impact cell morphology. However, by day 14 the cells were able to spread and migrate across all meshes forming dense cellular networks.

Cell metabolic activity for HDF and hADSCs demonstrates that all meshes support cell attachment and proliferation for up to 14 days (Figure 8b,c). The PCLSH meshes have comparable results to PCL only and PCLSH30 is the highest by day 14, although no significant difference is observed between samples. These results indicate that all meshes do not induce any cytotoxicity, including meshes with high SH concentrations. The high metabolic cellular activity, observed in meshes containing high SH concentration, might be explained by the higher hydrophilicity of the SH meshes, which can increase the attachment of serum proteins in the correct conformation, and the additional source of nutrients provided by SH.

High glucose levels have been shown to have a negative effect on cell proliferation [74,75]. However, the current results indicate no major variation in HDF and hADSCs proliferation and viability between the PCL only and glucose containing SH meshes. The PCLSH30 mesh has a high initial glucose content, but this rapidly decreases within 48 h due to degradation and dissolution of the SH component of the mesh (Figure 8c). The PCL samples show no presence of glucose at all timepoints.

Furthermore, the XPS analysis shows an increase in the oxygen level in the SH meshes. This is potentially a good indicator of the healing capabilities of the meshes as high levels of oxygen in the wound environment can improve the healing process through recruitment of cells and promoting cell attachment and spreading [76]. Further investigation is required to understand the role of oxygen in the SH containing meshes, determine the glucose oxidase concentration and how the presence of SH impacts cell behavior. Finally, the electrospun SH meshes all show cytocompatibility (for both HDF and hADSCs) up to 14 days. This is in agreement with other studies that show that the incorporation of honey is not cytotoxic [35,36,37].

### 3.8. Antibacterial Performance of Electrospun Meshes

The antibacterial properties of the PCLSH electrospun meshes were evaluated using a disk diffusion test against three different bacterial strains: *S. aureus, E. coli, and P. aeruginosa*. These strains represent both Gram-negative and positive bacteria that commonly infect humans and which an effective broad spectrum antibacterial should inhibit.

**Figure 9:**
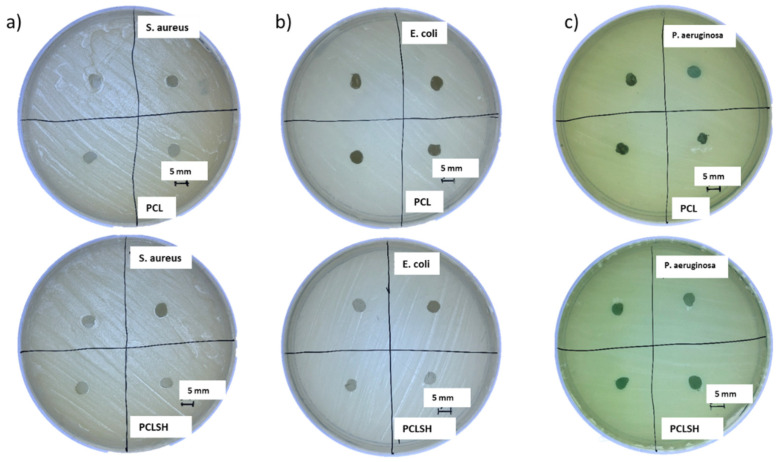
Inhibition zone of PCL (**top**) and PCLSH30 (**bottom**) electrospun meshes on agar plates using the disk diffusion method. (**a**) *S. aureus*, (**b**) *E. coli*, and (**c**) *P. aeruginosa* (scale = 5 mm).

Figure 9 presents the PCL and PCLSH30 meshes on the agar plates to investigate the bacterial inhibition zone. SH alone has previously been identified to have significant antibacterial properties [52,58,59]. However, the results show that PCLSH30 meshes were not able to prevent bacterial growth. PCLSH10 and PCLSH20 meshes were also tested but no inhibition zone was observed (images not shown). The H_2_O_2_ assay also showed no production of H_2_O_2_ in any of the meshes up to 48 h (Figure S5). Subsequently, the primary antibacterial attribute of SH, production of H_2_O_2_, is non-functional in the currently fabricated meshes. The lack of H_2_O_2_ production, despite the presence of glucose, may be a result of the slow degradation process of PCL delaying the release of SH from the fibers, or due to a negative effect of the solvent (acetic acid) on glucose oxidase in SH during processing. Consequently, further optimization is required in material development such as alternative solvents and polymers to obtain an electrospun mesh that can produce a sustained release of H_2_O_2_. 

## 4. Conclusions

This preliminary study demonstrates the fabrication and characterization of electrospun PCL meshes containing SH. Solution electrospinning was used to produce random nanofibrous meshes with fiber diameter, mesh density, and mesh roughness dependent on SH concentration. The SH was shown to be incorporated into the PCL polymeric matrix through FTIR and XPS. The presence of SH impacted the crystallization process as a lower degree of crystallinity was observed in PCLSH meshes. The addition of SH increased the hydrophilicity and degradation rate of the meshes enhancing the suitability of the PCLSH meshes for skin applications compared to PCL alone. The comprehensive investigation of material properties is necessary in the development of biomedical devices to allow for regulatory approval and optimization of material properties. Furthermore, the PCLSH meshes were shown to be biocompatible and non-cytotoxic when seeded with either HDFs or hADSCs. Cell metabolic activity and viability was comparable or better than PCL meshes alone.

Despite the negative antibacterial performance of the electrospun meshes and lack of observable H_2_O_2_ production, this study demonstrates an initial preliminary concept for the fabrication of a H_2_O_2_ releasing electrospun mesh. Furthermore, these results highlight the importance of quantifying H_2_O_2_ when utilizing honey-based materials to understand antibacterial properties.

However, as this is a preliminary study, further investigation is required to determine the functionality of glucose oxidase within the meshes and optimize processing and material selection (polymers and solvents) to allow successful incorporation of SH and subsequent production of H_2_O_2_. Furthermore, the anti-microbial efficacy of the further developed meshes must be established with the generation, concentration, and release profile of H_2_O_2_ evaluated. Additionally, the role of H_2_O_2_ in modulating cell behavior in the electrospun meshes must be assessed [55].

Electrospinning is a suitable technology to develop drug delivery and slow-release systems for pharmaceutical applications especially in localized treatment in patients. This initial study is a promising development for an electrospun honey containing mesh for use in skin tissue engineering and wound dressing applications.

## Data Availability

The data presented in this study are available on request from the corresponding author.

## Supplementary Materials

The following are available online at https://www.mdpi.com/article/10.3390/ma15010089/s1. Figure S1. The morphology of PCL electrospun meshes processed with different voltages, flow rates, and needle-collector distances (scale bar = 50 µm). Figure S2. The morphology of PCLSH10 electrospun meshes processed with different voltages, flow rates, and needle-collector distances (scale bar = 50 µm). Figure S3. The morphology of PCLSH20 electrospun meshes processed with different voltages, flow rates, and needle-collector distances (scale bar = 50 µm). Figure S4. The morphology of PCLSH30 electrospun meshes processed with different voltages, flow rates, and needle-collector distances (scale bar = 50 µm). Figure S5. The presence of hydrogen peroxide in the PCL and PCLSH30 electrospun meshes up to 48 h was not observed or was negligible. Additionally, no units of glucose oxidase were detected in any PCLSH samples at any time point (data not shown).
