# Supplementary material for: Preliminary Characterization of a Polycaprolactone-SurgihoneyRO Electrospun Mesh for Skin Tissue Engineering
Source: Materials (Basel). 2021 Dec 23;15(1):89. doi: 10.3390/ma15010089 (PMC8746156; doi:10.3390/ma15010089)
Supplement: Supplementary file 1 [file materials-15-00089-s001.zip › materials-1383503-supplementary.pdf]

Supplementary materials: Preliminary characterisation of a polycaprolactone-SurgihoneyRO electrospun mesh for skin tissue engineering

Enes Aslan [1,2], Cian Vyas [2], Joel Yupanqui Mieles [2], Gavin Humphreys [3], Carl Diver [4] and Paulo JDS Bartolo [2,5]*

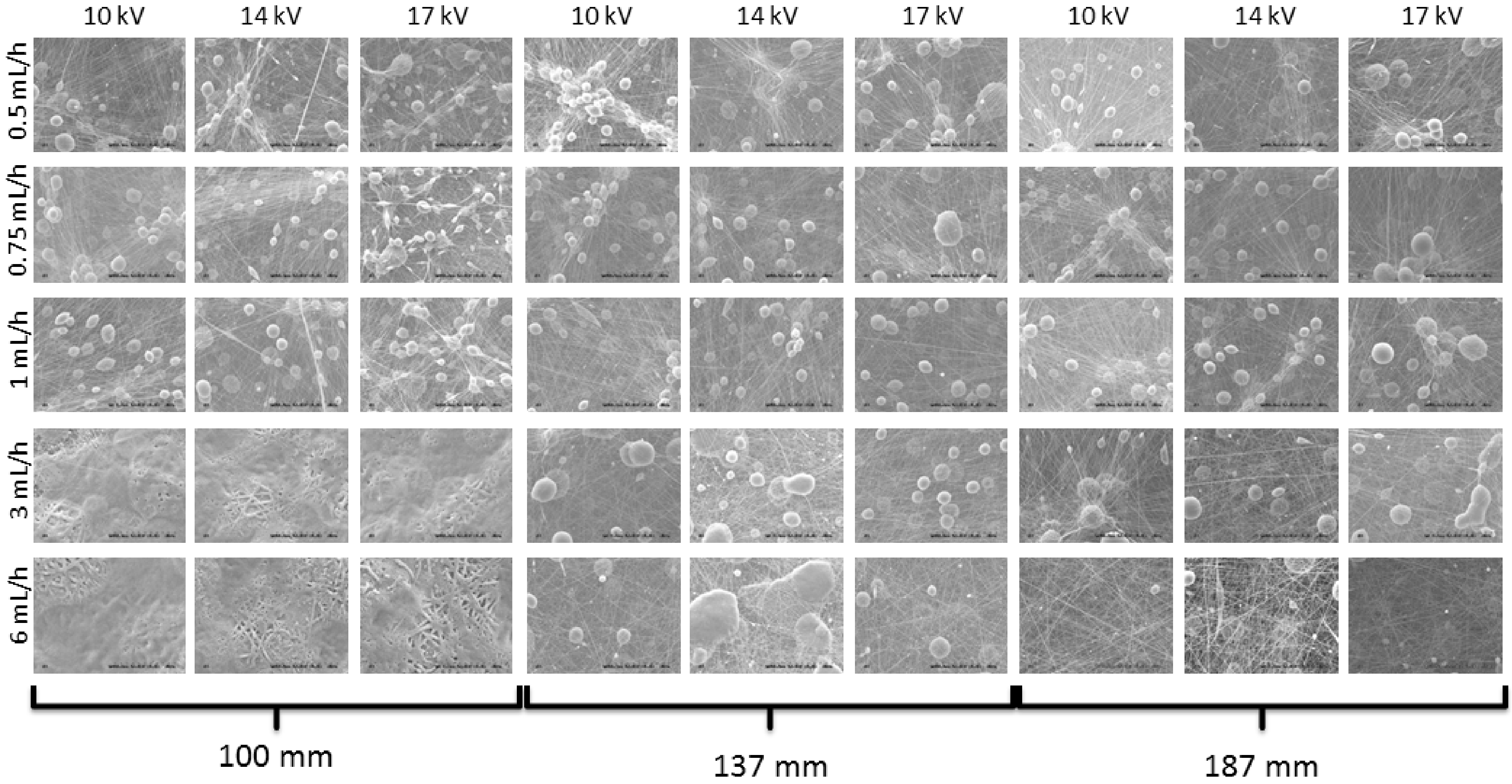

Figure S1. The morphology of PCL electrospun meshes processed with different voltages, flow rates, and needle-collector distances (scale bar = 50 µm).

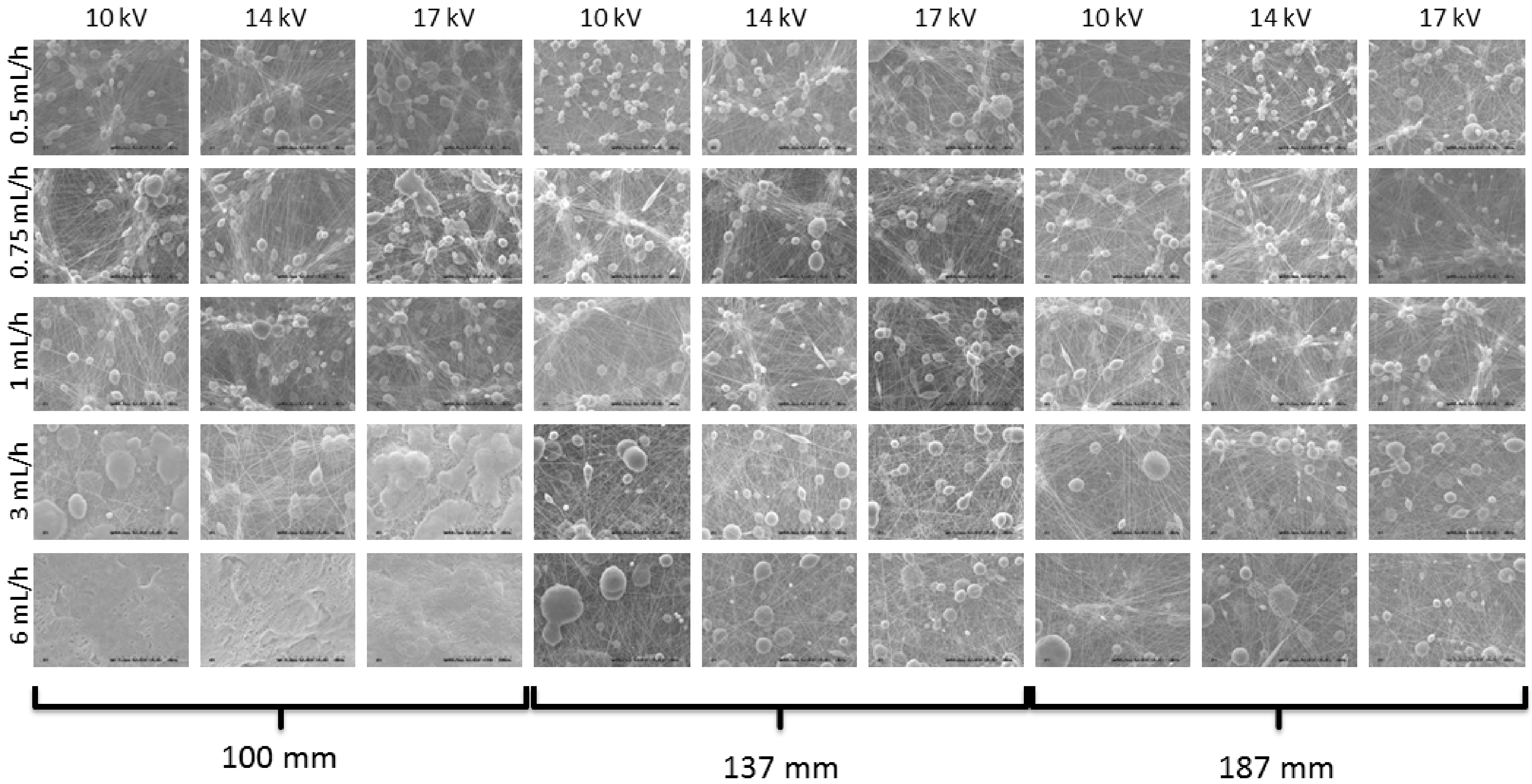

Figure S2. The morphology of PCLSH10 electrospun meshes processed with different voltages, flow rates, and needle-collector distances (scale bar = 50 µm).

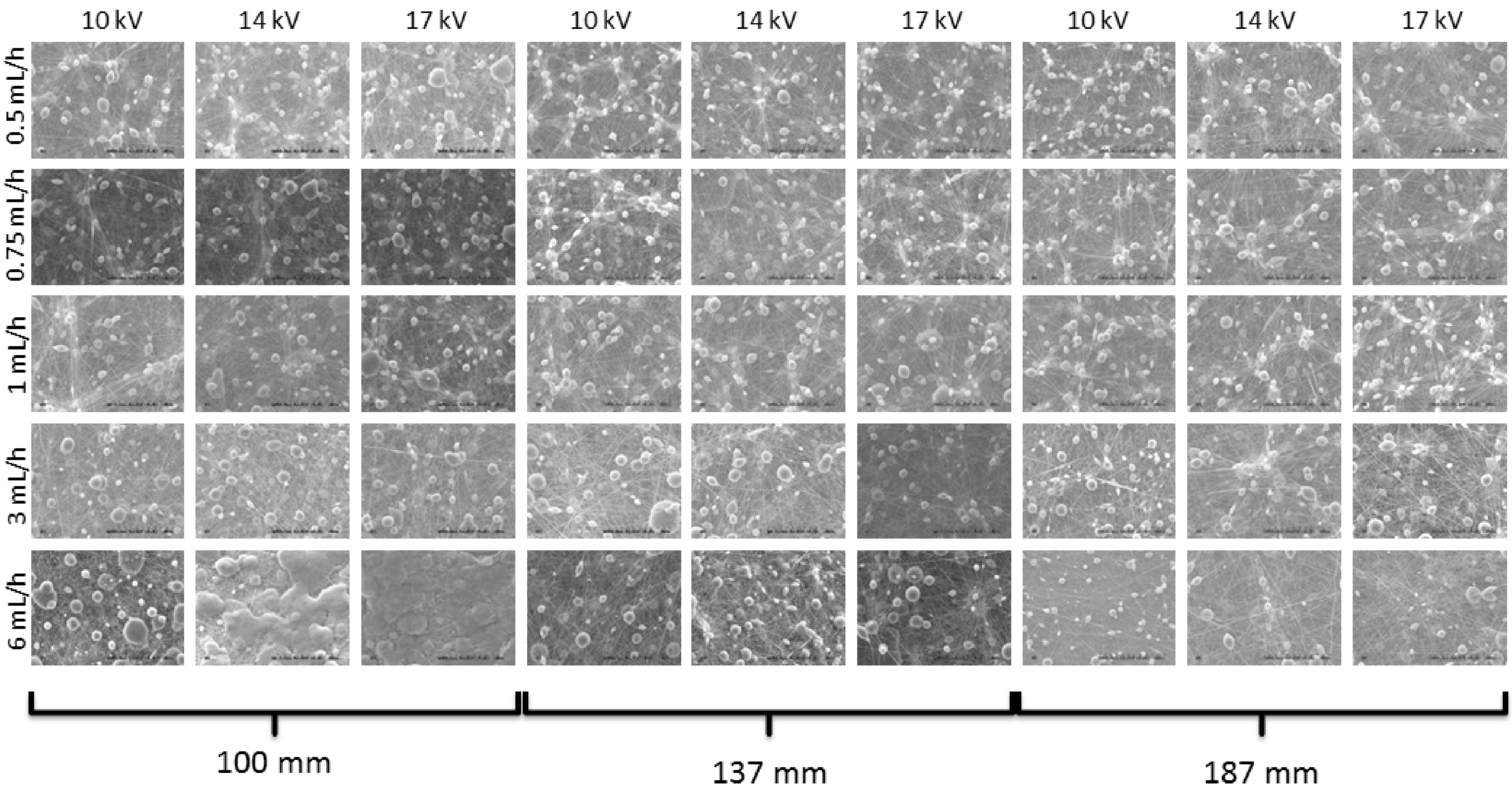

Figure S3. The morphology of PCLSH20 electrospun meshes processed with different voltages, flow rates, and needle-collector distances (scale bar = 50 μm).

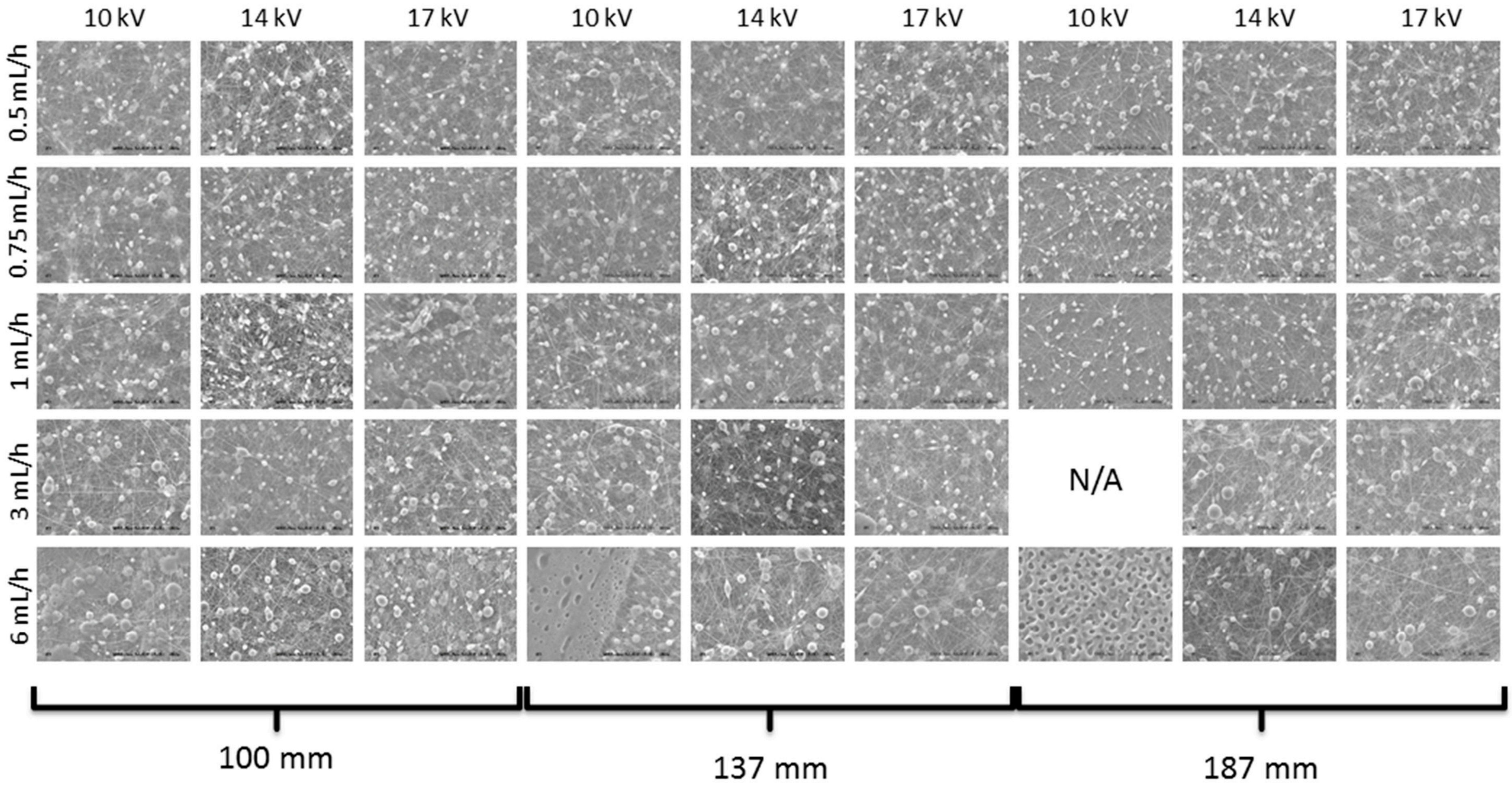

Figure S4. The morphology of PCLSH30 electrospun meshes processed with different voltages, flow rates, and needle-collector distances (scale bar = 50 μm).

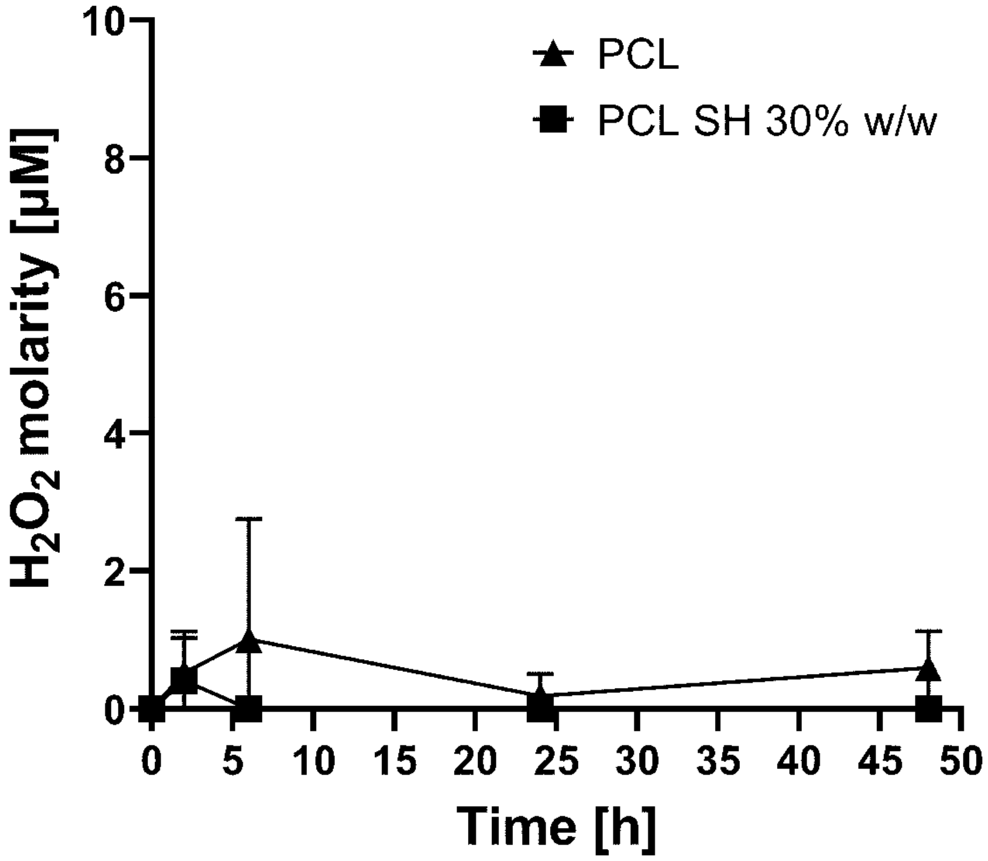

Figure S5. The presence of hydrogen peroxide in the PCL and PCLSH30 electrospun meshes up to 48 h was not observed or was negligible. Additionally, no units of glucose oxidase were detected in any PCLSH samples at any time point (data not shown).
